# Amount and Frequency of Alcohol Consumption and All-Cause Mortality in a Japanese Population: The JMS Cohort Study

**DOI:** 10.2188/jea.JE20081003

**Published:** 2009-05-05

**Authors:** Atsuko Sadakane, Tadao Gotoh, Shizukiyo Ishikawa, Yosikazu Nakamura, Kazunori Kayaba

**Affiliations:** 1 Department of Public Health, Jichi Medical University, Shimotsuke, Tochigi, Japan; 2 Wara National Health Insurance Clinic, Gujyo, Gifu, Japan; 3 Division of Community and Family Medicine, Center for Community Medicine, Jichi Medical University, Shimotsuke, Tochigi, Japan; 4 Saitama Prefectural University, School of Health and Social Science, Koshigaya, Saitama, Japan

**Keywords:** cohort studies, alcohol drinking, mortality, Japan

## Abstract

**Background:**

Lower mortality has been reported in light-to-moderate alcohol drinkers. We examined the association between the amount and frequency of alcohol consumption and all-cause mortality in a Japanese population.

**Methods:**

We conducted a prospective cohort study among 8934 Japanese people (3444 men and 5490 women) who completed a baseline survey between 1992 and 1995. We confirmed the date and cause of death by referring to death certificates. The Cox proportional hazards model was used to evaluate the effect of alcohol consumption on risk for all-cause mortality, after adjustment for potential confounding factors.

**Results:**

We identified 637 (397 men and 240 women) deaths during the 12.0 years of mean follow-up. Among men, as compared with non-drinkers, the relative risk was higher in ex-drinkers (hazard ratio [HR], 1.18), lower in light drinkers (HR, 0.95) and moderate drinkers (HR, 0.91), and significantly higher in heavy drinkers (HR, 1.67; 95% confidence interval, 1.10–2.55). Among women, light, moderate, and heavy drinkers were grouped into current drinkers. The relative risk was slightly higher in current drinkers (HR, 1.23), and that in ex-drinkers was near 1.0 (HR, 0.97). In stratified analysis, the harmful effects of heavy drinking were more severe among male smokers and younger men. In terms of frequency, men who drank only on special occasions had the highest mortality (HR, 1.28), regardless of alcohol intake per drinking session.

**Conclusions:**

In men, a near J-shaped association was identified between alcohol consumption and all-cause mortality. Both the amount and frequency of alcohol consumption were related to mortality.

## INTRODUCTION

Many studies have noted a J- or U-shaped association between alcohol consumption and all-cause mortality,^1^^–^^4^ ie, lower all-cause mortality was observed among light-to-moderate drinkers as compared to non-drinkers and heavy drinkers. These patterns of association are thought to be mainly due to a reduction in coronary heart disease^5^^,^^6^ among light-to-moderate drinkers, because alcohol increases high-density lipoprotein (HDL) cholesterol level,^7^ decreases platelet aggregation,^8^ and has beneficial effects on endothelial function and markers of inflammation.^9^^,^^10^ However, heavy drinkers are more likely to suffer from injuries, alcoholic liver disease, some types of cancers, hypertension, and cerebral hemorrhage, and thus have increased mortality.^11^

There has been some controversy as to whether the J- and U-shaped associations between alcohol consumption and mortality can be explained by the fact that former drinkers who have stopped drinking because of medical problems have been included in the category of non-drinkers (the “sick quitter” hypothesis).^12^ As a result of the higher mortality in non-drinkers, who comprise both life-time abstainers and former drinkers, the relative risk for mortality in light-to-moderate drinkers appears to be lower. However, some recent studies have observed J- or U-shaped associations even after separating former drinkers from never drinkers^2^; therefore, the sick quitter hypothesis alone is not sufficient to explain the lower mortality among light-to-moderate drinkers.

The usual or average amount of alcohol consumed per some predefined interval has been used in most epidemiologic studies as the measurement of drinking habits within study populations. In addition to the amount of alcohol consumed, measures such as consumption pattern^13^^–^^15^ and type of alcoholic beverage^16^ are believed to be related to several health consequences. Drinking pattern, which refers to the frequency of alcohol intake, the quantity of alcohol consumed per drinking occasion, or some combination of these measurements, is considered important because it is related to all-cause mortality, some cardiovascular diseases, and injuries, independent of the usual amount of alcohol consumed. In particular, episodic heavy drinking (binge drinking) has been found to increase the risk of coronary heart disease.^17^

In Japan, measures of alcohol consumption,^18^ eg, proportion of abstainers, proportion of heavy drinkers, and types of alcoholic beverages consumed, differ from those in Western countries.^19^ The proportion of abstainers lies between proportions reported in the United States and most European countries, the proportion of male heavy drinkers is higher, and more Japanese drinkers consume spirits (Japanese *sake* and *shochu*). In addition, mortality from coronary heart disease, which contributes to lower all-cause mortality among light-to-moderate drinkers, is considerably lower in Japan than in Western countries.^20^ Therefore, studies of Japanese populations may improve our understanding of the relationship between alcohol and mortality.

The objective of this study was to investigate the association between categories of alcohol consumption based on daily alcohol consumption and all-cause mortality in a middle-aged Japanese population that included men and women. Furthermore, we assessed the association between frequency of alcohol intake and all-cause mortality, the role of alcohol consumption in cause-specific mortality, and the effects of age and smoking status on the association between alcohol consumption and mortality.

## METHODS

### Study population

The Jichi Medical School Cohort Study is a multicenter study that investigates the risk factors for cardiovascular disease in Japanese populations.^21^ Baseline data were obtained between 1992 and 1995. The study participants are 12 490 Japanese individuals who underwent mass screening programs in 12 rural communities located across Japan. Among the 12 490 participants, we excluded individuals who refused to be followed up (*n* = 95), lived outside study areas at the baseline survey (*n* = 2), were younger than 40 years or older than 69 years (*n* = 1859), did not respond to questions about alcohol consumption (*n* = 1028), or had a past medical history of myocardial infarction, stroke, or neoplasm (*n* = 572). Consequently, 8934 (3444 men and 5490 women) were analyzed. The mean age of analyzed participants was 56.3 years in men and 56.4 years in women; among those not included in the analysis, mean age was 52.6 years in men and 52.4 years in women.

The study design and procedures were reviewed and approved by each municipal government and by the Ethics Committee for Epidemiological Research at Jichi Medical School. Written informed consent was obtained from all prospective participants.

### Alcohol intake

At baseline, information on alcohol consumption status, including non-drinkers, former drinkers, and current drinkers, was assessed by self-administered questionnaire. For current drinkers, frequency of alcohol intake was classified into 4 categories: almost every day, less than 3 days per week, less than 2 days per month, and on special occasions only. In addition, we requested information on the usual amount of alcohol consumed in 1 typical drinking session for each of 5 types of alcoholic beverages (Japanese *sake*, Japanese spirits, whisky, beer, and wine). We calculated daily alcohol consumption by summing alcohol content in each of these beverages multiplied by the index derived from frequency of alcohol intake (1 for almost every day, 0.3 for ≤3 days/week, 0.04 for ≤2 days/month, and 0.02 for only on special occasions). Participants were categorized into non-drinkers, ex-drinkers, light drinkers (≤22.9 g/day), moderate drinkers (23.0–68.5 g/day), and heavy drinkers (≥68.6 g/day) for men, and non-drinkers, ex-drinkers, and current drinkers for women. Because of the small percentages of moderate and heavy drinkers among women, it was not possible to use the same consumption categories for women and men. The categorization was based on the traditional Japanese unit of alcohol, *go*, which is equivalent to 22.8–22.9 g of alcohol. In addition, we categorized participants by frequency of alcohol consumption into non-drinkers, ex-drinkers, those who drank only on special occasions, those who drank ≤2 days/month, those who drank ≤3 days/week, and those who drank almost every day.

### Socioeconomic and behavioral profiles and laboratory data

Socioeconomic and behavioral variables that were considered to be potential confounding factors were ascertained by a standardized questionnaire, which comprised questions on age, marital status, education level, smoking status, and physical activity. In order to calculate body mass index (BMI), body weight and body height were measured. In addition, blood pressure was obtained using an automated sphygmomanometer. Total and high-density lipoprotein (HDL) cholesterol was measured, then low-density lipoprotein (LDL) cholesterol was calculated using the Friedwald equation.

Marital status was coded as currently married or unmarried. Education level was categorized into 2 strata (≤9 or ≥10 years). Smoking habits were classified as never smoker, ex-smoker, 1–19 cigarettes/day, or ≥20 cigarettes/day for men, and never smoker, ex-smoker, or current smoker for women. The physical activity index, which was developed in the Framingham Study, was calculated by totaling the hours at each level of activity within a day, and multiplying this by a weighting based on the oxygen consumption required for that activity. The index was categorized into 3 strata: low (≤28.4), medium (28.5–36.4), or high (≥36.5). BMI was categorized into 3 strata according to the classification of The Japan Society for the Study of Obesity (<18.5, 18.5–24.9, or ≥25.0 kg/m^2^).

### Follow-up and case ascertainment

The primary end point of the study was death from all causes. Date and cause of death were confirmed by referring to death certificates at public health centers in each community, with official approval. In addition, we contacted all participants annually by means of a health examination program in each community. For those who did not participate in the health examination, we obtained information on their health status by mail or telephone. The survival status of all the 8934 study participants was examined at least once. Among them, 211 participants left the study area after follow-up had begun, and follow-up was terminated at the time of relocation. Causes of death were coded according to the International Classification of Diseases, 10th Revision.

### Statistical analysis

Statistical analyses were performed separately for men and women. First, associations between socioeconomic and behavioral characteristics and alcohol consumption status were evaluated. Mean blood pressure and cholesterol level in each alcohol consumption category were also assessed. Then, we used the Cox proportional hazards regression model to assess associations between alcohol consumption and risk of mortality from all causes. We adjusted for age, education level, marital status, smoking status, physical activity index,^22^ and BMI. We also assessed the association between the frequency of alcohol consumption and all-cause mortality. Statistical analysis was performed using SPSS 15.0 J for Windows.

## RESULTS

Table 1 shows the proportions of non-, ex-, and current drinkers among the study participants. Current drinkers were categorized according to daily alcohol consumption. The proportions of the categories differed between men and women: there were more current and ex-drinkers among men. As mentioned above, because the proportions of moderate and heavy drinkers were so small in women, we grouped slight, moderate, and heavy drinkers into current drinkers for further analysis.

**Table 1.** Baseline characteristics of study participants by amount of alcohol consumed

	Non-drinkers	Ex-drinkers	Current drinkers			

			All	Light drinkers (≤22.9 g/day)	Moderate drinkers (23.0–68.5 g/day)	Heavy drinkers (≥68.6 g/day)
Men	*n* = 706	*n* = 119	*n* = 2619	*n* = 1202	*n* = 1195	*n* = 222
​ Age (%)						
​ ​ 40–49 years	17.2	1.5	81.3	37.4	33.6	10.4
​ ​ 50–59 years	19.2	2.5	78.3	35.4	35.9	7.1
​ ​ 60–64 years	20.4	5.2	74.5	33.3	36.5	4.7
​ ​ 65–69 years	26.7	5.2	68.1	33.1	32.3	2.7
​ Tobacco smoking (%)						
​ ​ Never smokers	25.6	2.7	71.7	40.7	27.5	3.4
​ ​ Ex-smokers	19.2	6.1	74.7	38.1	31.4	5.2
​ ​ ≤19 cigarettes/day	21.4	2.5	76.1	34.0	36.0	6.0
​ ​ ≥20 cigarettes/day	18.0	2.2	79.8	29.3	41.0	9.6
​ Education level (%)*						
​ ​ ≤9 years	21.6	3.8	74.6	33.4	35.5	5.8
​ ​ >9 years	19.5	3.1	77.4	36.3	34.0	7.1
​ Marital status (%)						
​ ​ unmarried	30.9	4.2	64.9	30.9	26.2	7.9
​ ​ married	19.8	3.4	76.8	35.3	35.1	6.4
​ Physical activity index (%)^†^						
​ ​ ≤28.4	22.5	4.9	72.6	34.5	29.5	8.6
​ ​ 28.5–36.4	20.2	3.6	76.2	35.2	35.3	5.7
​ ​ ≥36.5	19.9	2.5	77.6	34.9	36.8	5.9
​ Body mass index (%)						
​ ​ ≤18.4 kg/m^2^	31.6	2.9	65.4	31.6	30.9	2.9
​ ​ 18.5–24.9 kg/m^2^	20.1	3.1	76.8	34.7	35.5	6.7
​ ​ ≥25.0 kg/m^2^	20.3	4.4	75.3	36.2	33.2	5.9
​ Systolic blood pressure (mm Hg)^‡^	128.2 ± 19.5	126.1 ± 20.4	133.0 ± 20.2	130.7 ± 19.5	134.6 ± 20.1	137.0 ± 22.5
​ Diastolic blood pressure (mm Hg)^‡^	77.0 ± 11.9	75.7 ± 21.0	80.7 ± 12.1	79.3 ± 12.1	81.6 ± 11.9	83.5 ± 12.9
​ Total cholesterol (mg/dl)^‡^	187.1 ± 34.7	181.0 ± 32.2	186.5 ± 34.4	188.1 ± 33.6	184.7 ± 34.2	188.8 ± 38.8
​ HDL cholesterol (mg/dl)^‡^	43.2 ± 11.2	44.6 ± 11.2	48.9 ± 13.5	47.7 ± 12.6	53.0 ± 13.6	54.0 ± 15.8
​ LDL cholesterol (mg/dl)^‡^	119.0 ± 31.1	112.5 ± 26.6	112.1 ± 31.1	115.1 ± 30.2	106.3 ± 30.4	104.2 ± 34.2

Women	*n* = 4101	*n* = 68	*n* = 1321	*n* = 1237	*n* = 68	*n* = 16
​ Age (%)						
​ ​ 40–49 years	64.4	0.9	34.7	N.A.	N.A.	N.A.
​ ​ 50–59 years	73.9	1.1	25.1	N.A.	N.A.	N.A.
​ ​ 60–64 years	80.9	1.7	17.4	N.A.	N.A.	N.A.
​ ​ 65–69 years	81.4	1.4	17.2	N.A.	N.A.	N.A.
​ Tobacco smoking (%)						
​ ​ Never smokers	76.4	0.9	22.6	N.A.	N.A.	N.A.
​ ​ Ex-smokers	50.0	8.8	41.2	N.A.	N.A.	N.A.
​ ​ Current smokers	52.6	4.0	43.4	N.A.	N.A.	N.A.
​ Education level (%)*						
​ ​ ≤9 years	77.1	1.4	21.5	N.A.	N.A.	N.A.
​ ​ >9 years	71.9	1.1	27.0	N.A.	N.A.	N.A.
​ Marital status (%)						
​ ​ unmarried	72.7	3.7	23.6	N.A.	N.A.	N.A.
​ ​ married	74.8	1.1	24.1	N.A.	N.A.	N.A.
​ Physical activity index (%)^†^						
​ ​ ≤28.4	70.9	1.6	27.5	N.A.	N.A.	N.A.
​ ​ 28.5–36.4	76.1	1.2	22.7	N.A.	N.A.	N.A.
​ ​ ≥36.5	76.0	0.9	23.2	N.A.	N.A.	N.A.
​ Body mass index (%)						
​ ​ ≤18.4 kg/m^2^	75.8	1.2	23.0	N.A.	N.A.	N.A.
​ ​ 18.5–24.9 kg/m^2^	75.0	1.0	24.0	N.A.	N.A.	N.A.
​ ​ ≥25.0 kg/m^2^	74.1	1.7	24.2	N.A.	N.A.	N.A.
​ Systolic blood pressure (mm Hg)^‡^	129.0 ± 20.4	131.2 ± 19.8	127.8 ± 21.6	N.A.	N.A.	N.A.
​ Diastolic blood pressure (mm Hg)^‡^	76.8 ± 11.8	78.3 ± 12.1	76.7 ± 12.4	N.A.	N.A.	N.A.
​ Total cholesterol (mg/dl)^‡^	200.5 ± 34.2	199.8 ± 33.9	196.6 ± 33.3	N.A.	N.A.	N.A.
​ HDL cholesterol (mg/dl)^‡^	52.0 ± 12.2	50.6 ± 10.7	54.4 ± 12.9	N.A.	N.A.	N.A.
​ LDL cholesterol (mg/dl)^‡^	126.1 ± 31.3	125.5 ± 32.5	121.6 ± 29.8	N.A.	N.A.	N.A.

N.A.: Not available

*Length of education.

^†^Physical activity index refers to metabolic equivalent task-hours.

^‡^Mean ± standard deviation.

Table 1 also shows the association between socioeconomic and behavioral characteristics and alcohol consumption. Among men, older participants were likely to be non- or ex-drinkers, while younger men drank more heavily. Smoking status and alcohol consumption had similar distributions. There were more non-, ex-, and heavy drinkers among unmarried participants. Those who were not physically active were likely to be non-, ex-, or heavy drinkers. Obese participants were likely to be ex-, or light drinkers, and lean participants were less likely to drink. Among women, younger participants were more likely to be drinkers than older participants. As observed in men, the distribution of smoking status was similar to that of alcohol consumption in women. Participants with a higher education level were more likely to drink. More unmarried than married participants were ex-drinkers. Participants who were not physically active were more likely to drink than those who were physically active. Among men, systolic and diastolic blood pressure and HDL-cholesterol were positively associated with, and LDL-cholesterol was inversely associated with, alcohol consumption. Among women, systolic and diastolic blood pressure and total and LDL-cholesterol were higher, and HDL-cholesterol was lower, in non-drinkers than in current drinkers.

During the mean 12.0 years of follow-up, there were 637 deaths (397 men and 240 women). The follow-up rate was 99.2%. As shown in Table 2, among men the crude mortality rate was highest in ex-drinkers and lowest in light drinkers. Among women, the crude mortality rate did not differ between non-, ex-, and current drinkers.

**Table 2.** Number of deaths, person-years, and crude mortality rate by amount of alcohol consumed

	Non-drinkers	Ex-drinkers	Current drinkers			

			All	Light drinkers (≤22.9 g/day)	Moderate drinkers (23.0–68.5 g/day)	Heavy drinkers (≥68.6 g/day)
Men						
​ Number of deaths (all)	92	20	285	125	130	30
​ ​ Stroke	6	2	33	15	15	3
​ ​ Coronary heart disease	9	2	4	4	0	0
​ ​ Cancer	35	7	129	53	62	14
​ Person-years	8421	1329	31 295	14 373	14 345	2578
​ Crude mortality rate*	10.9	15.1	9.1	8.7	9.1	11.6

Women						
​ Number of deaths (all)	178	3	59	N.A.	N.A.	N.A.
​ ​ Stroke	25	1	4	N.A.	N.A.	N.A.
​ ​ Coronary heart disease	13	0	2	N.A.	N.A.	N.A.
​ ​ Cancer	73	2	30	N.A.	N.A.	N.A.
​ Person-years	49 469	783	16 088	N.A.	N.A.	N.A.
​ Crude mortality rate*	3.6	3.8	3.7	N.A.	N.A.	N.A.

N.A.: Not available

*per 1000 person-years.

The association between alcohol consumption and risk of all-cause mortality is shown in Table 3. In men, with non-drinkers as the reference, relative risk was slightly higher in ex-drinkers (hazard ratio [HR], 1.18; 95% confidence interval [CI], 0.71–1.96), slightly lower in light drinkers (HR, 0.95; 95% CI, 0.72–1.26) and moderate drinkers (HR, 0.91; 95% CI, 0.69–1.19), and significantly higher in heavy drinkers (HR, 1.67; 95% CI, 1.10–2.55). Excepting ex-drinkers, alcohol consumption and all-cause mortality showed a near J-shaped association. Among women, relative risk was slightly higher in current drinkers (HR, 1.23; 95% CI, 0.90–1.69), but not significantly so; relative risk in ex-drinkers was approximately 1.0 (HR, 0.97; 95% CI, 0.30–3.07). We adjusted only for age in the primary analysis, and for age, marital status, education level, smoking status, physical activity, and BMI in the subsequent analysis. Multivariate adjustment did not alter the associations. When we excluded the first 2 years of follow-up, the higher mortality risk in ex-drinkers disappeared, but the association between alcohol consumption and mortality in current drinkers did not change either among men or women.

**Table 3.** Age- and multivariate-adjusted relative risk of all-cause mortality by amount of alcohol consumed

	Non-drinkers	Ex-drinkers	Current drinkers			

			All	Light drinkers (≤22.9 g/day)	Moderate drinkers (23.0–68.5 g/day)	Heavy drinkers (≥68.6 g/day)
Men						
​ Age-adjusted	1	1.21 (0.75–1.96)^†^	0.96 (0.76–1.21)	0.91 (0.69–1.19)	0.92 (0.71–1.21)	1.67 (1.10–2.54)
​ Multivariate-adjusted*	1	1.18 (0.71–1.96)	0.98 (0.71–1.96)	0.95 (0.72–1.26)	0.91 (0.69–1.19)	1.67 (1.10–2.55)

Women						
​ Age-adjusted	1	0.97 (0.31–3.05)	1.26 (0.93–1.69)	N.A.	N.A.	N.A.
​ Multivariate-adjusted*	1	0.97 (0.30–3.07)	1.23 (0.90–1.69)	N.A.	N.A.	N.A.

N.A.: Not available

*Adjusted for age, tobacco smoking, education level, marital status, body mass index, and physical activity index.

^†^Hazard ratio (95% CI).

Regarding cause of death, 43.1% of male deaths and 43.8% of female deaths were from cancer; cardiovascular diseases (stroke or coronary heart disease) accounted for 14.1% of male deaths and 18.8% of female deaths. Among men, risk of death from stroke or coronary heart disease was responsible for increasing all-cause mortality in ex-drinkers. In light and moderate drinkers, risk of death from coronary heart disease was lower, whereas that from stroke increased. In male heavy drinkers, the risk of death from stroke or cancer increased, but that from coronary heart disease did not, as compared with male non-drinkers. In women, the number of cause-specific deaths in ex- and current drinkers was so small that we were not able to analyze cause-specific mortality risk.

In stratified analysis by smoking status (smoked or not at baseline), risk of all-cause mortality in non-smoking men was highest in ex-drinkers (HR, 1.88; 95% CI, 1.02–3.45) and lowest in heavy drinkers (HR, 0.78; 95% CI, 0.28–2.21). Among male current smokers, the risk was lowest in ex-drinkers (HR, 0.52; 95% CI, 0.16–1.67) and highest in heavy drinkers (HR, 2.13; 95% CI, 1.32–3.43). Ninety-two percent of female participants were non-smokers; therefore, stratification by smoking status did not affect the association between alcohol consumption and all-cause mortality in women. Furthermore, when we performed analysis stratified by age (40–59 vs 60–69 years), there were more current drinkers in the younger subgroup among both men and women. Among the younger subgroup, mortality risk was approximately 20% higher in male heavy drinkers and 30% higher in female current drinkers, as compared to the older subgroup. However, mortality risk in male light and moderate drinkers did not differ between age subgroups.

Table 4 shows the frequency of alcohol consumption among current drinkers. Approximately 73% of male current drinkers drank almost every day, whereas the proportions of infrequent drinkers—those who drank only on special occasions or ≤2 days/month—were small. Among female current drinkers, the proportions of these categories did not differ greatly. The association between frequency of alcohol consumption and all-cause mortality is shown in Table 5. Relative risk in men was highest in those who drank only on special occasions (HR, 1.28; 95% CI, 0.71–2.32), regardless of alcohol intake per drinking session. In contrast, among women, relative risk was highest in those who drank almost every day, although we were not able to adjust for alcohol intake because of statistical limitations.

**Table 4.** Frequency of alcohol intake among current drinkers*

	Current drinkers				
	
	All	Special occasion	≤2 days/month	≤3 days/week	Almost every day
Men					
​ Number	2619	134	189	382	1914
​ (%)	(100)	(5.1)	(7.2)	(14.6)	(73.1)

Women					
​ Number	1321	234	340	405	342
​ (%)	(100)	(17.7)	(25.7)	(30.7)	(25.9)

*Categories for never drinkers and ex-drinkers are shown in Table 1.

**Table 5.** Multivatiate-adjusted relarive risk of all-cause mortality by frequency of alcohol intake

	Non-drinkers	Ex-drinkers	Current drinkers			
	
			Special occasion	≤2 days/month	≤3 days/week	Almost every day
Men						
​ Model 1*	1	1.18 (0.71–1.97)^‡^	1.19 (0.69–2.07)	0.80 (0.48–1.34)	1.07 (0.74–1.55)	0.97 (0.75–1.25)
​ Model 2^†^	1	1.19 (0.69–2.08)	1.28 (0.71–2.32)	0.78 (0.46–1.33)	1.01 (0.68–1.52)	0.87 (0.64–1.18)

Women						
​ Model 1*	1	0.96 (0.30–3.04)	1.24 (0.63–2.44)	1.11 (0.63–1.96)	1.07 (0.61–1.90)	1.50 (0.92–2.43)

*Adjusted for age, tobacco smoking, education level, marital status, body mass index, and physical activity index.

^†^Adjusted for variables in model 1 and alcohol consumption per drinking session (this model did not fit women).

^‡^Hazard ratio (95% CI).

## DISCUSSION

In our prospective cohort study of a Japanese population, a near J-shaped association was identified between alcohol consumption and all-cause mortality among men. Light and moderate drinkers consuming ≤68.5 g alcohol per day had a slightly lower relative risk, while heavy drinkers consuming ≥68.6 g had a significantly higher relative risk, as compared to non-drinkers. Among women, the relative risk for current drinkers was slightly, but not significantly, higher. Analysis of the association between the frequency of alcohol consumption and all-cause mortality revealed that men who drank only on special occasions and women who drank almost every day had the highest mortality risk.

The results of our study in men were consistent with several earlier studies of alcohol consumption and mortality, including prospective studies and meta-analyses. According to the updated meta-analysis conducted by Di Castelnuovo et al,^23^ low levels of alcohol consumption (20–40 g/day in men and 10–20 g/day in women) were associated with lower all-cause mortality. With respect to Japanese studies, in both the Japan Public Health Center-based prospective study on cancer and cardiovascular diseases (JPHC Study)^24^ and the Japan Collaborative Cohort Study for Evaluation of Cancer Risk (the JACC Study),^25^ the association between alcohol consumption and all-cause mortality was J-shaped. Although our study was not able to determine the exact amount of alcohol consumption that was related to low mortality, these 2 studies identified alcohol consumption categories with the lowest all-cause mortality: current drinkers consuming 1–149 g/week in the former study and those consuming 0.1–22.9 g/day in the latter study. In contrast, Nakaya et al reported a dose–response relationship between alcohol consumption and all-cause mortality.^26^ Relative risks of all-cause mortality in men were 1.10, 1.17, 1.16, and 1.62 in those consuming <22.8, 22.8–45.5, 45.6–68.3 and ≥68.4 g/day, respectively.

With respect to frequency of consumption, it is possible that consuming alcohol only on special occasions is related to hazardous drinking patterns such as binge drinking, especially in men, although we were not able to identify this pattern of consumption with our questionnaire. Those men who drank only on special occasions consumed 45.9 g alcohol per session on average, which was similar to that consumed by daily drinkers (47.6 g). In women, consumption was 19.2 g for those who drank only on special occasions, and 22.4 g for daily drinkers. Several studies have observed that infrequent alcohol consumption is associated with higher mortality than frequent consumption, even if the average alcohol consumption was the same.^15^ Other studies have investigated the effects of occasional heavy drinking on mortality, regardless of the usual amount of alcohol consumed, and have found higher mortality with this drinking pattern.^14^

Although we distinguished former drinkers from non-drinkers (lifetime abstainers), a near J-shaped association in men was observed, which suggests that such an association cannot be explained only by the sick quitter hypothesis. Former drinkers had an approximately 20% higher risk of mortality than non-drinkers. After we excluded the first 2 years of follow-up, the higher mortality risk in former drinkers disappeared, but the associations between alcohol consumption and mortality in other categories were unchanged. Similarly, some investigators have noted that the U-shaped relationship between alcohol consumption and all-cause mortality remains after excluding the first 2 years of follow-up.^27^^,^^28^ These findings indicated that former drinkers had some subacute or chronic, rather than acute, illnesses or disorders. Therefore, we excluded participants with a history of stroke, myocardial infarction, or cancer at baseline. Although the details of their medical problems are unknown, we asked former drinkers the reasons for ceasing their alcohol consumption. About 55% of male former drinkers and 25% of female former drinkers answered that they quit because of medical problems.

Among our study participants, mortality from coronary heart disease was quite low (0.4 per 1000 person-years among men, 0.2 per 1000 person-years among women), which might have resulted in a relatively smaller reduction in all-cause mortality in light and moderate drinkers among men. In male heavy drinkers, the risk of death from stroke or cancer increased, but the risk of coronary heart disease did not. These results are partially consistent with those of other studies conducted in Japan.^29^ In a cohort study of male Japanese physicians, as compared with non-drinkers, the relative risk of all-cause mortality was 10% lower in light drinkers and 30% higher in heavy drinkers. In light drinkers, the risk of mortality from coronary heart disease was lower, but that from stroke and cancer was higher. Among heavy drinkers, increased mortality from cancer, stroke, and liver cirrhosis was identified. However, even among heavy drinkers, mortality from coronary heart disease was lower.

When we stratified participants by smoking status, mortality risk was higher in non-smoking ex-drinkers and heavy drinkers who smoked. The higher risk observed among non-smoking ex-drinkers can be explained by the same process as sick quitters hypothesis, as ex-smokers were more likely to stop drinking (Table 1). The harmful effects of heavy drinking were more severe in smokers, which was consistent with the findings of earlier studies.^5^^,^^24^ In addition, younger participants who drank heavily had higher mortality than older participants who drank heavily. Light-to-moderate drinking was not associated with greatly decreased mortality in older participants, although the majority of prospective studies^3^^,^^30^ and a meta-analysis^31^ have suggested that older persons do receive benefits from light-to-moderate alcohol consumption.

The advantages of our study were that the participants comprised both men and women, and that we were able to distinguish ex-drinkers from non-drinkers. In addition, we evaluated the alcohol consumption of study participants in terms of 2 different measurements: daily consumption and frequency, which allowed us to understand that both quantity and frequency have important effects on mortality. At baseline, we obtained data on past medical history, demographic characteristics, behavioral characteristics, and laboratory data such as cholesterol level, and this information enabled us to exclude participants who suffered from severe illnesses and to consider the effect of various confounding factors on the association between alcohol consumption and mortality. Furthermore, the follow-up rate was extremely high, and the mortality data (date and cause of death) were confirmed by referring to death certificates.

Our study had some limitations. Alcohol consumption, as estimated by the use of self-administered questionnaires, has not been validated. Sampling error, recall bias, and the perceived social desirability of certain behaviors, for example, might affect responses to questionnaires and lead to incorrect estimation of alcohol consumption. Feunekes et al recommended the use of a number of markers that could help determine whether misreporting of alcohol consumption has occurred.^32^ In our study, blood pressure and HDL-cholesterol were positively associated, and LDL-cholesterol was inversely associated, with alcohol consumption in men. Both of these findings were consistent with the known effects of alcohol intake on cardiovascular risk factors, which suggests that our assessment tool is acceptable, at least in grading participants by alcohol consumption.^33^ In addition, the proportions of participants assigned to each alcohol drinking status did not appreciably differ between the present study and a study conducted in Japan by Nakaya et al,^26^ in which the validity of their questionnaire was evaluated. The proportions of non-drinkers, ex-drinkers, and current drinkers in their study were 15.9%, 7.0%, and 77.1% (20.5%, 3.5%, and 76.0% in our study) in men and 72.1%, 4.0%, and 23.9% (74.4%, 1.2%, and 24.1% in our study) in women, respectively. These findings also support the use of our questionnaire. Participants were recruited through health check-up programs; therefore, their concern for their health may exceed that of the general population, which could result in differences in alcohol consumption and other behavioral profiles. Another selection bias might arise because 3556 (28%) individuals were excluded from the 12 490 participants. In addition, there were small numbers of moderate and heavy drinkers among women; therefore, we were not able to assess the association between mortality and alcohol consumption in women. Furthermore, we were not able to assess the association between alcohol consumption and cause-specific mortality in women because the numbers of cause-specific deaths in ex- and current drinkers were very small. Also, we did not consider changes in alcohol consumption status over time after enrolment. The British Regional Heart Study revealed that more participants reported non- or light drinking, and that fewer reported moderate or heavy drinking, as they were followed up.^34^ By contrast, Giovannucci et al, in The Nurses’ Health Study, observed a significant correlation between alcohol consumption at baseline and that assessed 4 years later.^35^

In conclusion, we observed that, as compared to male non-drinkers, mortality was slightly lower among male light drinkers and moderate drinkers, and higher among male heavy drinkers. The harmful effects of heavy drinking were more severe in younger participants and smokers. In addition, men who drank only on special occasions had a higher mortality risk. Although many studies have assessed the effects of alcohol consumption on health-related issues, further epidemiologic studies are needed because alcohol availability, individual preferences, and the characteristics of alcohol-drinking populations continue to change.
